# Inter- and Intrastate Variation of Mental Health Requirements for Assisted Living in Seven States

**DOI:** 10.1093/geroni/igab046.2032

**Published:** 2021-12-17

**Authors:** Sarah Dys

**Affiliations:** Portland State University/Vital Research, Oregon, United States

## Abstract

Little is known about states’ approaches to regulating mental health (MH) services in assisted living (AL) settings. Yet, one in nine AL residents are diagnosed with serious mental illness (Hua et al, 2020). This study describes the MH regulatory requirements in AL regulations within Arkansas, Louisiana, New Jersey, New York, Oklahoma, Pennsylvania, and Texas. Using health services regulatory analysis (Smith et al, 2021), we reviewed 2018 regulations for the 45 identified AL licenses within these states sourced from Nexis Uni. We summarize 16 MH requirements related to admission, care transitions, resident assessment, third-party services access, and staff training. Each state explicitly addressed at least one of the identified MH requirements, though few states have consistency across all AL types within a state. The most commonly addressed requirements related to admission limitations, assessment, and transfer to psychiatric units. Understanding these requirements promotes a holistic approach to practices that meet residents' needs.

